# Papillary thyroid cancer and cutaneous melanoma: An often underrecognized pair

**DOI:** 10.1016/j.jdcr.2024.05.026

**Published:** 2024-05-28

**Authors:** Michelle Chernyak, Dana Hutchison, Janellen Smith

**Affiliations:** Department of Dermatology, University of California, Irvine, Irvine, California

**Keywords:** BRAF V600E, cutaneous metastasis, melanoma, papillary thyroid carcinoma

## Introduction

Papillary thyroid carcinoma (PTC) is a relatively common endocrine malignancy; however, cutaneous metastases are exceedingly rare—found in less than 1% of all cases—and often signal widespread metastatic disease.^1^ Malignant melanoma (MM) is the fifth most common cancer in the United States with incidence rising.^2^ Interestingly, the *BRAF* V600E mutation is the most frequent genetic alteration in both PTC and MM.^2^ Here, we present a rare case of cutaneous metastatic *BRAF* V600E PTC in a patient with a history of MM. Our findings contribute to a growing body of evidence supporting a potential association between these 2 conditions.

## Case report

**Fig 1:**
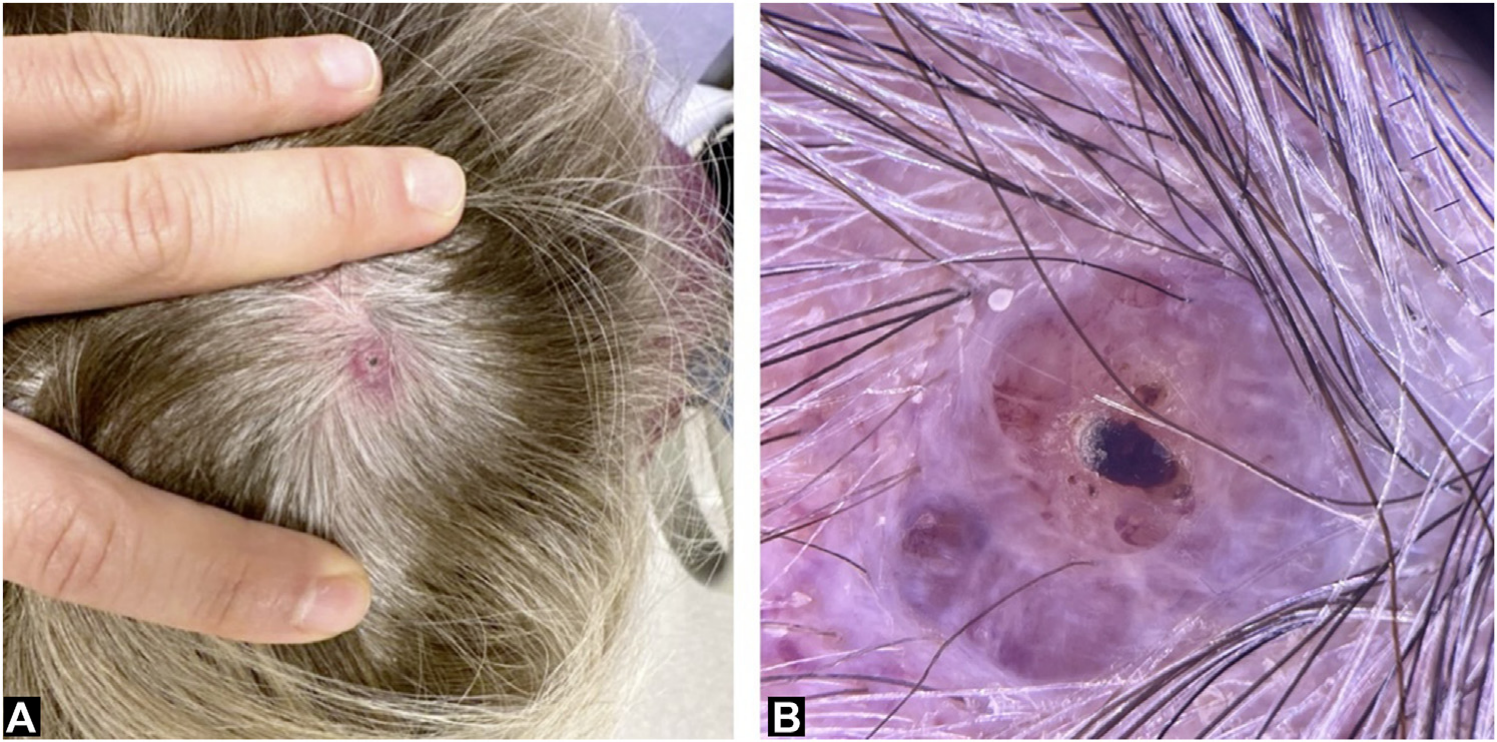
**A,** Physical examination revealed an erythematous papule with hemorrhagic crust. **B,** Milky red and purple clods on dermatoscopy.

A 79-year-old Caucasian woman sought evaluation for an enlarging, nonpainful, erythematous to violaceous papule on her scalp present for the past 3 months (Fig 1). Her medical history included MM on the right calf, with a 0.3-mm Breslow depth, surgically excised 6 years prior. She had been diagnosed with *BRAF* V600E–positive PTC, which had metastasized to the lungs 4 years prior to her current presentation. Treatment involved ablative measures with radioactive iodine and total thyroidectomy. In addition, the patient underwent several courses of kinase inhibitors, including VEGF inhibitor lenvatinib, MEK inhibitor trametinib, and BRAF inhibitor dabrafenib, each of which were discontinued owing to poor tolerance. Notably, she had discontinued treatment with trametinib and dabrafenib 10 months before her presentation to dermatology.

**Fig 2:**
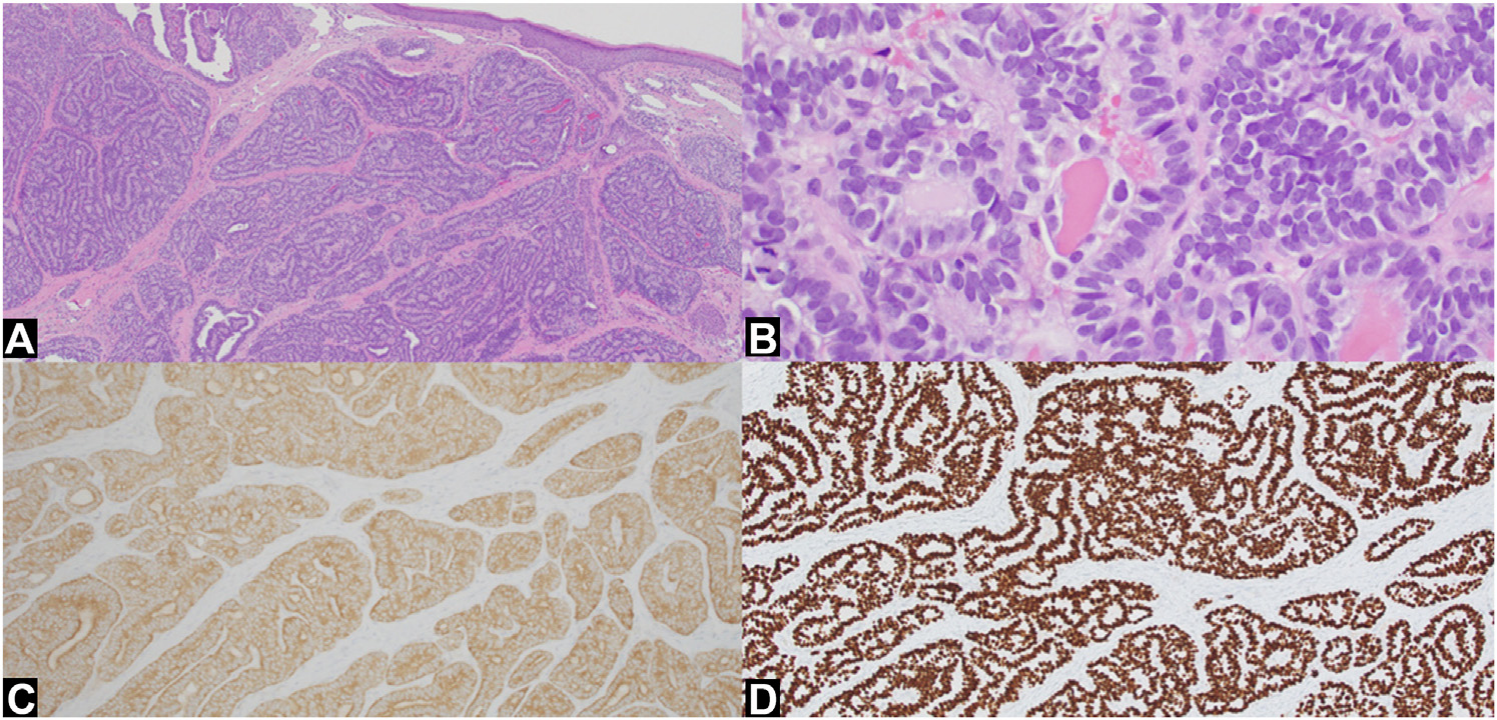
**A,** Tumoral involvement of the dermis with papillated follicles lined by cuboidal epithelial cells. **B,** Fronds of atypical cells surrounding bright pink colloid material. **C,** Diffusely positive BRAF V600E staining. **D,** Positive immunohistochemical staining for thyroid transcription factor-1. (**A** and **B,** Hematoxylin-eosin stain; original magnifications: **A,** ×4; **B,** ×40.)

Shave biopsy of the scalp lesion revealed metastatic PTC (Fig 2). Laboratory workup revealed elevated carbohydrate antigen 19-9 and thyroglobulin levels (91 U/mL and 1513 ng/mL, respectively) and suppressed thyroid-stimulating hormone (0.01 mIU/L). Computed tomography revealed innumerable scattered pulmonary nodules, bony lesions, and pancreatic lesions concerning for metastatic disease. A nodular lesion in the left thyroid bed was enlarged compared with prior imaging. Magnetic resonance imaging of the brain revealed a metastatic focus in the right temporal lobe. Given her extensive disease burden, the patient ultimately opted not to pursue further therapy and was lost to follow-up.

## Discussion

Cutaneous metastasis of PTC is exceptionally rare, manifesting in less than 1% of PTC cases and signaling a poor prognosis.^1^ PTC most commonly metastasizes to the cervical lymph nodes and less frequently to the lungs, bone, liver, and brain.^3^ Dermal metastases of PTC emerge 2 to 20 years after initial diagnosis, presenting as indolent erythematous or violaceous dermal nodules or plaques. They may present asymptomatically or have associated tenderness, pruritus, or ulcerations.^1^ The head and neck are the most common anatomical sites for cutaneous metastasis, with 66% of cases involving the scalp.^1^ The clinical differential diagnosis for scalp lesions with milky red and purple clods includes amelanotic melanoma, highlighting the importance of histopathology in making this diagnosis.

*BRAF* mutations have been implicated in 36% to 69% of PTC cases.^4^ First identified in 2002, the BRAF mutation has been associated with various malignancies, most commonly melanoma, followed by PTC and colorectal carcinoma.^2^ One study analyzed 4460 patients diagnosed with PTC and 14,569 with cutaneous melanoma between 1966 and 2011 and found that patients with PTC have a 1.8-fold increased risk for developing melanoma.^5^ Further, patients with preexisting melanoma have a 2.3-fold increased risk of developing PTC.^5^ This may be in part due to their shared *BRAF* mutation.^5^

The wild-type *BRAF* gene encodes a nonreceptor serine/threonine-specific protein kinase that acts on the mitogen-activated protein kinase (MAPK) pathway to promote cellular differentiation and survival.^2^ When missense mutations occur, the MAPK pathway is upregulated, resulting in oncogenesis through increased cell division and proliferation.^4^ The presence of *BRAF* mutations in certain PTC subtypes results in especially aggressive phenotypes and has been linked to distant metastasis, recurrence, and advanced tumor stage.^4^ Common therapeutic strategies for thyroid malignancies include radioactive iodine treatment; however, cases of PTC that express the *BRAF* mutation have been associated with decreased avidity to radioiodine.^6^ In thyroid malignancies, the *BRAF* mutation has been linked to decreased expression of iodide-handling genes for radioiodine ablation (such as the sodium iodine transporter, thyroglobulin, and thyroid peroxidase), potentially diminishing treatment efficacy.^6^

Interestingly, both melanoma cells and thyroid cancer cells express the thyroid-stimulating hormone receptor. When stimulated, thyroid-stimulating hormone receptor activates the phosphoinositide 3-kinase and Akt/protein kinase B (PI3K/AKT) pathway, working concomitantly with the MAPK pathway toward tumorigenesis.^6^ One study found that isolated *BRAF* inhibition in cancer cells leads to the overexpression of the receptor tyrosine kinase c-Met.^7^ This, in turn, causes reactivation of both the MAPK pathway and PI3K/AKT pathway, indicating that dual inhibition may be necessary for effective treatment.^7^ One study demonstrated that synchronous suppression of both the MAPK pathway and PI3K/AKT pathway induced G_0_/G_1_ cell cycle arrest and subsequent melanoma cell apoptosis, offering a potential therapeutic strategy.^6^ In addition, concurrent suppression of these pathways induces expression of thyroid iodide-handling genes (sodium iodine transporter, thyroglobulin, and thyroid peroxidase) within melanoma cells, allowing for the uptake of radio iodide and potentially indicating radioiodine ablation therapy as an adjuvant to current melanoma treatment.^6^

Beyond the MAPK pathway, *BRAF* mutations have been implicated in the upregulation of Snail, a zinc finger transcriptional factor, which downstream promotes decreased expression of E-cadherin.^8^ This aids in the epithelial-mesenchymal transition, which allows for malignant cells to detach from their site of origin, resulting in metastasis.^8^ This transformation has also been noted in PTC and correlates with lymph node involvement.^8^

MM is often treated with a combination of BRAF inhibitors and MEK inhibitors.^9^ Such a combination is more efficacious than just targeting *BRAF* alone.^9^ Owing to the shared mutations in PTC, it may be beneficial to employ a similar therapeutic approach.^9^ One study demonstrated in 9 patient-derived PTC organoid models that dual pathway inhibition offered a synergistic effect that decreased cell viability to a greater extent compared with monotherapy.^9^ The use of BRAF inhibitor monotherapy, such as vemurafenib and dabrafenib, in PTC can lead to resistance due to upregulation of a downstream functional enzyme involved in DNA repair, redox factor-1 (Ref-1), requiring synchronous inhibition of Ref-1 with E3330 to increase vemurafenib sensitivity.^10^ Studies have noted that Ref-1 expression is decreased early in tumorigenesis of melanoma, and later disease stages associated with metastasis present with increased expression of Ref-1.^10^ This expression may be implicated in varying responses to *BRAF* inhibitions between PTC and MM.^10^

We present a rare case of relapsing PTC metastatic to the skin in a patient with a history of MM. Heightened clinical awareness regarding the potential link between PTC and *BRAF*-positive MM is recommended because a delayed diagnosis can significantly increase patient morbidity and mortality. Given the significant crossover in genomic mechanisms of carcinogenesis, it may be pertinent to screen for thyroid nodules via physical examination in patients with a history of *BRAF*-positive melanoma. Proactive monitoring and timely intervention can substantially improve outcomes for patients facing these malignancies.

## Conflicts of interest

None disclosed.
